# Proteome-wide characterization of PTMs reveals host cell responses to viral infection and identifies putative antiviral drug targets

**DOI:** 10.3389/fimmu.2025.1587106

**Published:** 2025-05-30

**Authors:** Xiaolu Li, Adam Kabza, Ashley N. Ives, Julianne Thiel, Katrina M. Waters, Wei-Jun Qian, Amy C. Sims, Tong Zhang

**Affiliations:** ^1^ Biological Sciences Division, Pacific Northwest National Laboratory, Pacific Northwest National Laboratory, Richland, WA, United States; ^2^ Environmental Molecular Sciences Laboratory, Pacific Northwest National Laboratory, Richland, WA, United States; ^3^ Hanford High School, Richland, WA, United States; ^4^ Nuclear Chemical & Biological Technologies Division, Pacific Northwest National Laboratory, Richland, WA, United States

**Keywords:** PTMs, viral infection, antiviral drug, proteome, phosphorylation, redox, ubiquitination, acetylation

## Abstract

Post-translational modifications (PTMs) are biochemical modifications that can significantly alter protein structure, function, stability, localization, and interactions with other molecules, thereby activating or inactivating intracellular processes. A growing body of research has begun to highlight the role of PTMs, including phosphorylation, ubiquitination, acetylation, and redox modifications, during virus-host interactions. Collectively, these PTMs regulate key steps in mounting the host immune response and control critical host pathways required for productive viral replication. This has led to the conception of antiviral therapeutics that focus on controlling host protein PTMs, potentially offering pathogen-agnostic treatment options and revolutionizing our capacity to prevent virus transmission. On the other hand, viruses can hijack the host cellular PTM machinery to modify viral proteins in promoting viral replication and evading immune surveillance. PTM regulation during virus-host interactions is complex and poorly mapped, and the development of effective PTM-targeted antiviral drugs will require a more comprehensive understanding of the cellular pathways essential for virus replication. In this review, we discuss the roles of PTMs in virus infection and how technological advances in mass spectrometry-based proteomics can capture systems-level PTM changes during viral infection. Additionally, we explore how such knowledge is leveraged to identify PTM-targeted candidates for developing antiviral drugs. Looking ahead, studies focusing on the discovery and functional elucidation of PTMs, either on the host or viral proteins, will not only deepen our understanding of molecular pathology but also pave the way for developing better drugs to fight emerging viruses.

## 1 Introduction

Viruses are obligate intracellular parasites that are dependent on living host cells to mediate all stages of the virus replication cycle and transmission (1, 2). Successful viral replication starts with the attachment and entry of a virus into a host cell. Once inside the host cell, viruses use cellular machinery to synthesize viral proteins which are required to replicate viral genomic DNA/RNA. Nascent viral genomes and structural proteins are assembled into progeny viruses and released from the host cell to start a new infection cycle. The host can mount a series of defensive responses including recognition of viral DNA/RNA, activation of both innate (e.g., interferon response and natural killer cell activation) and adaptive immune responses (viral antigen presentation and T cell and B cell activation), and the inflammatory response (3, 4). Accordingly, investigation of the molecular basis of virus-host interactions has elucidated key steps in viral pathogenesis and evolution between viruses and their hosts. In addition, such knowledge is also crucial for developing novel host directed antiviral strategies.

To understand the complex interplay between viruses and their host cells, past studies have identified key interacting components during various steps of a viral infection. In the case of severe acute respiratory syndrome coronavirus 2 (SARS-CoV-2), the causative agent of COVID-19, the scientific community extensively studied interactions between the viral spike glycoprotein and the cellular receptor angiotensin-converting enzyme 2 (ACE2) (5). Protein-protein interactions (PPIs) or protein-RNA interactions at other steps including uncoating (6), genome replication (7, 8), and particle production (9) have also been extensively studied for viruses from the majority of virus families. Biochemical approaches such as co-immunoprecipitation and bimolecular fluorescence complementation allow researchers to focus on specific proteins of interest and have resulted in comprehensive profiling of the viral-host PPI network (10). Collectively, these data provide tens of thousands of experimentally validated viral-human PPIs, leading to an unprecedented depth of understanding of virus-host interactions (11). Besides PPIs, transcriptome analysis provides a mechanistic understanding of viral-host interactions by generating information on the viral sequences and host cell pathways that are responsive to viral infection (12). Similar to transcriptomics, proteomics also enables system-wide analysis of cellular processes during viral infection (13).

Post-translational modifications (PTMs) add another layer of complexity to the regulatory mechanisms in virus-host interactions. PTMs are covalent additions of functional groups to specific amino acid residues that can significantly alter the structure and function of a protein. Around 600 types of PTMs have been described (14), and several reversible PTMs including phosphorylation, ubiquitination, acetylation, glycosylation, methylation, and redox modifications have been extensively studied in cell signaling and the pathophysiology of human diseases. In the context of virus-cell interactions, the importance of PTMs has been well-demonstrated (15, 16), and the outcome of PTM regulation can be either promoting or inhibiting viral replication. For instance, the human immunodeficiency virus (HIV) transactivator Tat protein binds and activates the cellular cyclin-dependent kinase 7 (CDK7), which in turn phosphorylates and activates RNA polymerase II, leading to a higher level of viral replication (17). In contrast, activation of interferon-induced protein kinase R (PKR) by many viruses inhibits viral replication by reducing the synthesis of viral proteins (18). To characterize PTMs that are important in cellular defense mechanisms against viral infection globally, PTM proteomics approaches have been applied and generated a growing list of viral infection-specific PTM events (19, 20). PTM proteomics is a specialized branch in proteomics designed to enrich and quantify tens of thousands of proteins with PTMs. Compared to traditional proteomics approaches that measure protein abundance, PTM proteomics can capture the kinetics of PTM changes following infection and thus provide a better mechanistic understanding of critical virus-host interactions (21).

In this review, we will provide examples of how PTMs regulate virus replication, including how PTMs on specific host or viral protein(s) change their function and contribute to the outcome of transmission and disease severity (Section 2). We will focus on phosphorylation, ubiquitination, acetylation, and redox modifications (oxidation of the thiol group of cysteine residues). The first three PTMs have been extensively studied in a variety of viral infection models using mature analytical approaches (22–24). Little is known about protein thiol oxidation during viral infection, however it is well-established that virus infection causes oxidative stress, and thus it is likely that thiol oxidation will be important in viral infections. While Section 2 discusses PTM events on specific proteins, most of which were studied using biochemical and genetical approaches, the rest of the review focuses on recent progress using PTM proteomics to better understand virus host interactions. We provide a primer on both bottom-up (Section 3) and top-down (Section 4) PTM proteomics approaches for non-experts. We then present the PTM landscape and insights gained in both host cell proteomes (Section 5) and viral proteins (Section 6) by summarizing recent PTM proteomics studies. We conclude with a discussion on how the PTM mechanisms can be used for targeted drug development.

## 2 PTM mechanisms in host-viral interactions

In this section, we discuss the biochemical mechanisms by which PTMs mediate changes in protein function and ultimately influence the outcome of viral infection. While extensive studies have been conducted on many proteins, we present selected examples from current literature to illustrate several well-characterized impacts of PTMs: structural changes, alterations in protein–protein interactions, and regulation of protein localization, stability, and enzymatic activity. These mechanisms are typically elucidated using conventional biochemical and genetic approaches. Studies employing omics-based methods will be discussed in Sections 5 and 6.

### 2.1 Phosphorylation

**Figure 1:**
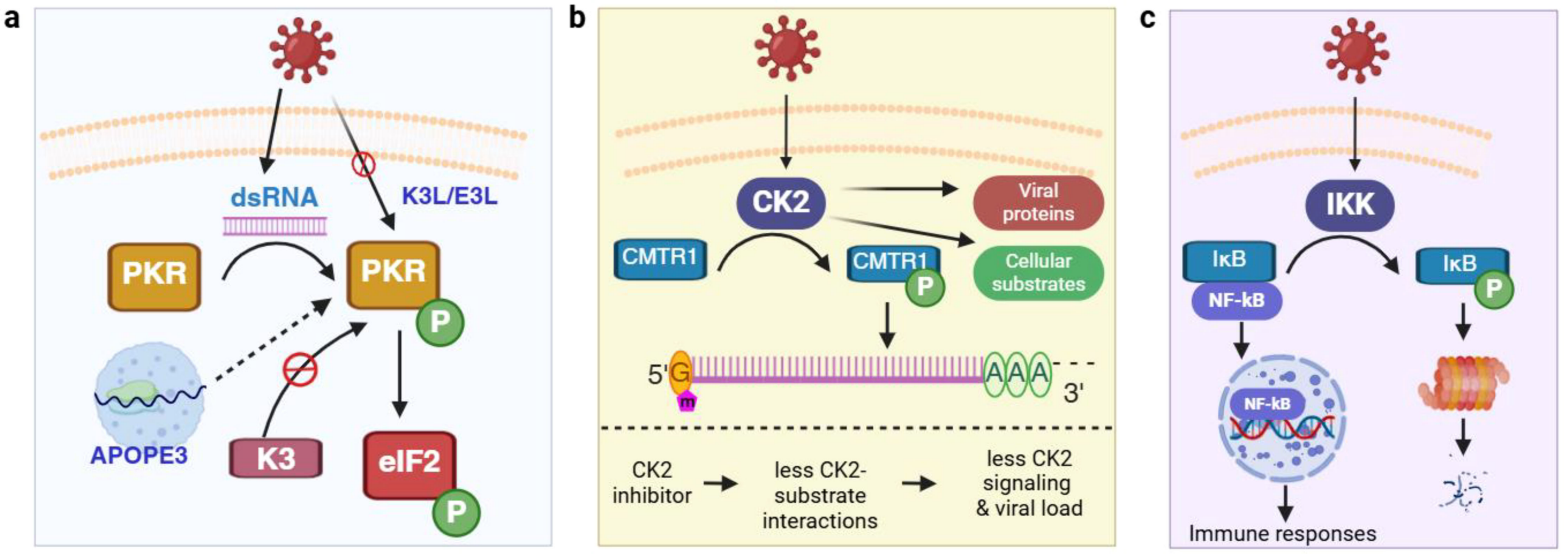
Protein kinases and phosphorylation in mediating host-virus interaction. **(a)** Control of translation initiation via the regulation of PKR activity; **(b)** CK2 phosphorylates multiple host cell substrates (e.g., CMTR1) and viral proteins (e.g., N) and is a drug target to control viral infection. **(c)** Viral infection actives NF-κB signaling via phosphorylation. APOPE3, apolipoprotein B mRNA-editing enzyme catalytic polypeptide-like 3; CK2, casein kinase II; CMTR1, cap methyltransferase 1; dsRNA, double-stranded RNA; eIF2α, eukaryotic translation initiation factor 2; IκB, inhibitor of kappa B; IKK, IκB kinase; N, viral nucleocapsid protein; NF-κB, nuclear factor kappa-light-chain-enhancer of activated B cells; PKR, dsRNA-dependent protein kinase. Green circle with white letter P denotes phosphorylation. This and all the following figures were created using BioRender (https://www.biorender.com/).

Protein phosphorylation is catalyzed by protein kinases. One of the best-characterized protein kinases in antiviral responses is double-stranded RNA (dsRNA)-dependent protein kinase (PKR) (18). A broad range of RNA and DNA viruses produce dsRNA during replication, which intracellular PKR can sense (**Figure 1a**). This activates PKR via autophosphorylation, and the activated PKR phosphorylates eukaryotic translation initiation factor 2 (eIF2α) to inhibit translation initiation in infected cells. After the shutdown of host cell translation, untranslated mRNAs (including viral RNA) and RNA-binding proteins are stored in transient cytoplasmic organelles called stress granules (SG) (25). Some SG proteins play important roles in regulating PKR activity, as recently demonstrated for the A3B member of the apolipoprotein B mRNA-editing enzyme catalytic polypeptide-like 3 (APOBEC3) family (26). The significant role of the PKR pathway in host cellular defense can also be appreciated by the evolution of multiple anti-PKR mechanisms in multiple virus strains. Vaccinia virus encodes K3L and E3L, which mimic and sequester dsRNA to prevent PKR activation (3). Similarly, poxviruses produce a mimic of eIF2 called K3 as a pseudosubstrate that can inhibit PKR activity, and the inhibitory potential of K3 varies considerably among even closely related virus species (27). Herpes simplex virus type 1 counteracts the impact of PKR activation by recruiting protein phosphatase 1, which de-phosphorylates and reactivates eIF2α (28).

Viruses depend on the host’s cellular metabolism for replication and can manipulate host metabolic pathways. 5’-AMP-activated protein kinase (AMPK) is a master regulator of cellular energy homeostasis and its activation promotes ATP production. Because virus replication is an energy intensive process, it is no surprise to see the activation of AMPK by many viruses including human cytomegalovirus (HCMV) (2), white spot syndrome virus (29), porcine reproductive and respiratory syndrome virus (30), and influenza virus (31). These viruses may require AMPK activation for successful infection, as knocking down AMPK or inhibiting its activity suppresses viral replication (29). However, this is not universal for all viruses as AMPK activation dampens the infection of Rift Valley fever virus (32) and flaviviruses (33).

Casein kinase II (CK2) is a Ser/Thr kinase that has been extensively studied in cancerous cells. In the context of viral infection, CK2 is activated by several viruses including SARS-CoV-2 and can directly bind to the nucleocapsid (N) protein (**Figure 1b**) (19). Accordingly, an increase in phosphorylation on many pathways downstream of CK2 such as cytoskeleton organization have been found upon infection. Although hundreds of CK2 substrates have been described, new ones are still being discovered. One recently found CK2 substrate is cap methyltransferase 1 (CMTR1), which methylates the first ribonucleotide of newly transcribed pre-mRNA (called an RNA cap) and enhances RNA stability and expression (34). CMTR1 is phosphorylated at 15 Ser/Thr sites and mutation of all sites to Ala results in a significantly lower level of influenza virus infection (35). Thus, CK2 can promote influenza replication by phosphorylating CMTR1, which in turn enhances RNA cap formation. Because of the broad substrate specificity and its prominent role in viral infection, CK2 has been proposed as an antiviral therapeutic target. Indeed, treating bovine kidney cells with CIGB-325, a synthetic peptide binding to CK2 substrates at the conserved phosphor-acceptor site, leads to a reduction in bovine coronavirus replication (36).

Besides PKR, AMPK, and CK2, many other kinases are also regulated during virus infection. Inhibitor of kappa B kinase (IKK), which phosphorylates and promotes the degradation of inhibitor of kappa B (IκB), is activated by numerous viruses (37, 38). This releases nuclear factor kappa-light-chain-enhancer of activated B cells (NF-κB) from binding with IκB, allowing NF-κB translocation to the nucleus and activates NF-κB-mediated immune responses. The mitogen-activated protein kinase (MAPK) pathway functions downstream of pattern recognition receptor (PRRs) in virus infection and regulates multiple immune responses including the development of cytokine storm (39, 40). Protein Kinase B (PKB, also known as Akt) can be activated in the early stages of herpes simplex virus 1 infection, which blocks apoptosis by phosphorylating and inhibiting proapoptotic factors in infected cells (41, 42). Activation of these kinases triggers the phosphorylation of various substrates, forming interconnected signaling networks.

Besides intracellular proteins, protein kinases can interact with viral proteins. Non-structural protein 1 (NS1) of Influenza A virus (IAV) is phosphorylated at the late stages of viral infection (43). Unphosphorylated NS1 plays an important role in depressing the host immune response by sequestering viral RNA from the cellular sensor retinoic acid-inducible gene I (RIG-I) and thus deactivating the innate immune response and interferon pathway (44). Phosphorylation at Thr49 impairs the binding of NS1^Thr49^ with viral RNA and abolishes its activity in inhibiting the host immune response (43). Thus, phosphorylation of NS1 switches off its interferon antagonistic activity at the late stage of viral infection. Examples listed here are just a few of the thousands of phosphorylation events during host-virus interaction, highlighting the importance of phosphorylation and the need for a high-throughput omics approach to characterize phosphoproteins at the proteome scale during viral entry, replication, and release.

### 2.2 Ubiquitination

**Figure 2:**
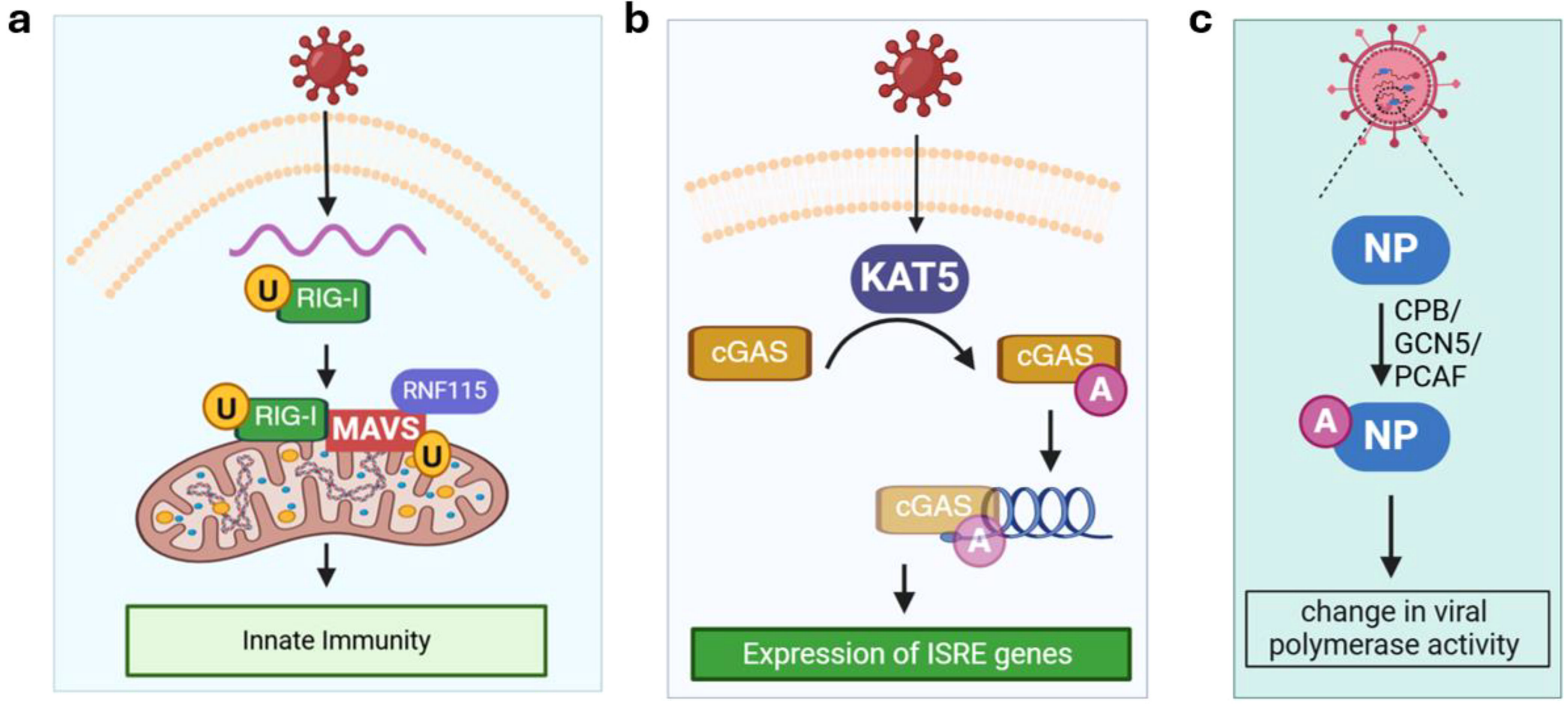
Ubiquitination and acetylation. **(a)** Ubiquitination of RIG-I and MAVS activates innate immunity. **(b)** Acetylation of cGAS promotes its binding with viral DNA and stimulates the expression of ISRE genes under viral infection (e.g., herpes simplex virus 1); **(c)** Acetylation of viral NP by different host cell acetyltransferases at distinct sites may lead to increase or decrease in viral polymerase activity. cGAS, mitochondrial antiviral-signaling protein; CPB, cAMP-response element–binding protein; GCN5, acetyltransferases general control non-repressed 5 protein; ISRE, interferon-stimulated response element; KAT5, lysine acetyltransferase 5; MAVS, mitochondrial antiviral-signaling protein; NP, non-structural protein; PCAF, P300/CBP-associated factor. RIG-I, retinoic acid-inducible gene I; RNF115, RING finger protein 115. Orange circle with black letter U denotes ubiquitination. Purple circle with white letter A denotes acetylation.

Ubiquitination defines the reversible addition of ubiquitin which regulates various cellular processes including protein degradation, trafficking, signal transduction, and transcriptional regulation during viral infection (16). The broad involvement of ubiquitination can be appreciated by the fact that the ubiquitin-proteasome pathway is heavily regulated during viral infection, which affects both viral replication and host defense mechanisms. RIG-I is a sensor of viral RNA and ubiquitination of RIG-I^K172^ in Sendai virus-infected HEK293T cells was reported almost 20 years ago (45). Such virus-induced ubiquitination is critical for binding of RIG-I to mitochondrial antiviral-signaling protein (MAVS), a key adaptor protein mediating cellular antiviral responses, and inducing type I interferon (IFN)-mediated innate immunity (**Figure 2a**). Since then, ubiquitination of an increasing number of both host cellular and viral proteins have been discovered. For instance, a recent study showed that MAVS is ubiquitinated by the E3 ligase RING finger protein 115 (RNF115, also called BCA2 and Rabring7) (46). In this ubiquitination-based molecular control of MAVS activity, MAVS interacts with RNF115 constitutively in uninfected cells and K48-linked ubiquitination of MAVS leads to its degradation. RNA virus (e.g., encephalomyocarditis virus EMCV) infection promotes RIG-I–MAVS interaction, thereby releasing MAVS from RNF115 to activate immune signaling (47, 48). Other E3 ligases serve as antiviral effector proteins that can restrict viral infection by ubiquitinating viral proteins and promoting their degradation. One example is tripartite motif-containing protein 7 (TRIM7), which targets the membrane-remodeling protein 2BC of coxsackievirus for ubiquitination (49).

### 2.3 Acetylation

The role of acetylation in viral infections is emerging as many host proteins are acetylated during infection including RIG-I (50), TANK-binding kinase 1 (TBK1) (51), cGMP-AMP synthase (cGAS; a sensor of cytosolic DNA) (52). In most studies, the acetylation level of a host protein is altered by changing the levels of lysine acetyltransferases (KATs) or lysine deacetylases (KDACs) expression, and the impact on immune response and viral infection is assessed to establish a functional role for acetylation. Due to the existence of multiple KATs/KDACs, a screening assay is usually performed to identify the specific enzyme for a substrate protein of interest. For instance, overexpressing KAT5, but not other KATs, leads to acetylation of cGAS in HEK293 cells infected with herpes simplex virus 1 (**Figure 2b**). KAT5-catalzyed acetylation of cGAS enhances its binding activity toward DNA and promotes the expression of genes harboring an interferon-stimulated response element (ISRE).

Acetylation of viral proteins has been reported and summarized in a recent review (15). Among them, the IAV nucleoprotein (NP), which is functionally similar to eukaryotic histones and has been shown to be acetylated by cAMP-response element–binding protein (CBP), acetyltransferases general control non-repressed 5 protein (GCN5), and P300/CBP-associated factor (PCAF, **Figure 2c**) (53). Interestingly, acetylation on different sites of IAV NP showed the opposite effect on the viral polymerase activity (53). This suggests that acetylation of viral proteins can either bolster or hinder viral infection, depending on the substrate protein and site.

### 2.4 Redox modifications

**Figure 3:**
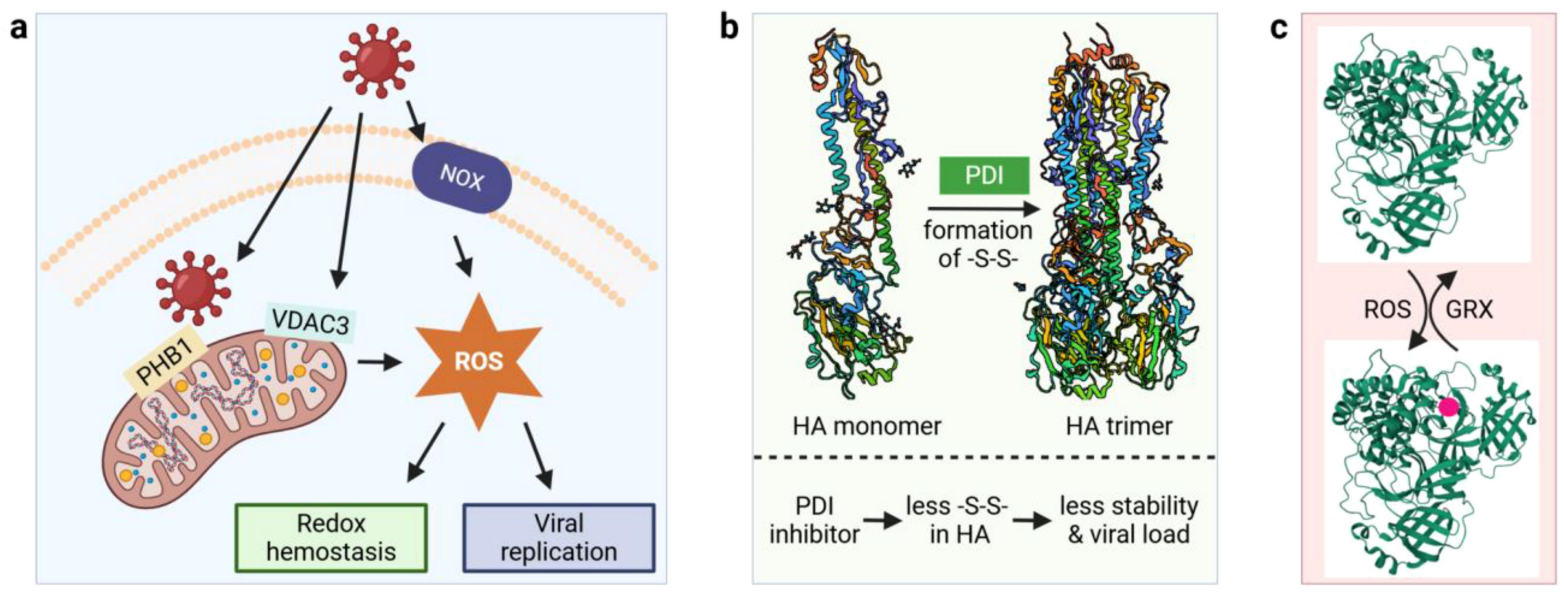
Virus-induced ROS generation and redox modifications. **(a)** Virus interacts with mitochondria and NOX to generate cellular ROS; **(b)** Oxidative folding of Influenza virus A viral HA protein by host PDIs. **(c)** Reversible glutathionylation of M^pro^. GRX, glutaredoxin; HA, hemagglutinin; M^pro^, main protease; NOX, NADPH oxidase; PDI, protein disulfide isomerase; PHB1, mitochondria-bound prohibitin 1; ROS, reactive oxygen species; -S-S-, disulfide bond; VDAC3, voltage-dependent anion channel. 3D structures were obtained from the protein data bank (PDB) with the following accession numbers: 3AL4 for HA monomer, 3LZG for HA trimer, and 2BX4 for M^Pro^. Red circle denotes glutathionylation.

Oxidative stress has been demonstrated to play a major role in virus-host interactions (54, 55). Mitochondria, in addition to synthesizing ATP to fuel the cell’s functions, also produce the majority of intracellular reactive oxygen species (ROS) and viruses have evolved a variety of mechanisms to hijack mitochondrial ROS production to promote viral replication. Enterovirus A71 replication complex proteins interact with mitochondria-bound prohibitin 1 (PHB1) leading to the disruption of mitochondrial integrity (**Figure 3a**) (56). Other viral proteins induce the production of ROS by manipulating ion channel activities, including the dysregulation of anionic channels by HIV Tat protein (57) and voltage-dependent anion channel (VDAC3) by hepatitis B virus X protein (58). In addition, ROS can be generated enzymatically by NADPH oxidase (NOX) during infection (59, 60). Virus-induced ROS production can lead to decreased glutathione (GSH) levels and activation of the antioxidant defense system including catalase (CAT) and superoxide dismutase (SOD1), as seen in COVID-19 patients (61). In fact, disruption of the redox balance in host cells seems to be a common theme during virus replication as hepatitis C and B (62) and IAV have also been shown to alter the host redox state (55). A disrupted redox balance can cause damage to a variety of cellular components leading to protein carbonylation, lipid oxidation, and DNA damage, which has been extensively reviewed elsewhere (63, 64). In contrast, viruses may benefit from excessive ROS as they utilize several mechanisms that exploit the oxidative environment (65). We will highlight how viruses use redox regulation for protein folding, interaction with host cell receptors and how they escape the detrimental impacts of ROS in this subsection.

Oxidative folding of viral proteins by the host cellular machinery illustrates one mechanism by which viruses exploit redox regulation for their benefit. IAV hemagglutinin (HA) is a homotrimeric glycoprotein and the viral attachment protein responsible for the binding of IAV to host cell surface and the agglutination of red blood cells (hence the name). The HA trimer is formed by oxidative folding of the monomers via disulfide bonds, which is catalyzed by protein disulfide isomerases (PDIs, **Figure 3b**). The PDI protein family consists of more than 20 members in mammals, and they catalyze both the formation and the isomerization of disulfide bonds in target proteins (66). An *in vitro* study found that ERp57, a PDI protein lacking a C-terminal ER retention motif, plays a major role in promoting the formation of HA trimers (67). To test the *in vivo* role of PDI in host-viral protein interactions, PDIA3 was knocked out in mouse epithelial cells because this member of the PDI family has a higher specificity for glycoproteins (68). Compared to wild-type mice, the mutant showed a significant decrease in viral burden (68). In addition, PDIA3 directly interacts with HA, and treatment of lung epithelial cells with LOC14, a PDI inhibitor, causes a decrease in the disulfide bond formation of HA. Thus, these data indicate that PDIA3 is important for HA trimer formation and that when the HA trimer is structurally more stable then IAV can infect naïve cells more easily. PDI also mediates the folding of E1 and E2 glycoproteins of hepatitis C virus (69) and the pre membrane protein of dengue virus (70).

As cysteine residues are the key amino acid in redox regulation, viral proteins rich in cysteine are good candidates for redox-active switches. The coronavirus spike glycoprotein (S, ~1250 amino acids in length) has 37–40 cysteine residues with an average of 3% occurrence, which is higher than the 1% cysteine in lower organisms (71). There are nine cysteine residues in the receptor binding domain (RBD) of the SARS-CoV-2 S protein (72). They form three structural disulfide bridges and one disulfide (Cys480-Cys488) that interacts with the host cell surface receptor ACE2. The amino acid composition within the Cys480-Cys488 loop is expected to affect the loop configuration and its ability to bind the receptor and thus infectivity. On the host cell side, ACE2 has a ferredoxin-like fold domain, and its expression is associated with oxidative stress (73). Importantly, in SARS-CoV-2 permissive species the ACE2 dimer has a disulfide bond, and that disulfide bond is absent in species impervious to the virus. Although the molecular details have not been delineated yet, these data imply redox mechanisms govern the interaction between the S protein and ACE2.

Another emerging redox regulation mechanism involves the reversible oxidation of critical cysteine residues of viral proteins under oxidative stress. The main protease (M^pro^) of SARS-CoV-2 is responsible for the cleavage of viral replicase polyproteins and is essential for viral replication (74). Active M^pro^ exists as a dimer and glutathionylation of Cys^300^ at the dimer interface inhibits the dimerization and enzymatic activity. Glutathionylation on non-functional monomers can be reversed by glutaredoxin (GRX), suggesting that reversible glutathionylation can regulate the activity of M^pro^ and protect the enzyme from over-oxidation under oxidative stress (**Figure 3c**) (75). In addition to glutathionylation, a very recent *in vitro* study demonstrated the formation of a sulfur-oxygen-nitrogen-oxygen-sulfur (SONOS) bridge between Cys^22^, Cys^44^ and Lys^61^ within M^pro^ (76). A SONOS bridge is the cross-linking of two cysteine thiols with one oxidized lysine, which has been observed in many pathogenic viral proteins and is believed to maintain structural stability under oxidative stress (77). Together, these redox switches may function by preventing the over-oxidation of M^pro^ catalytic Cys117.

## 3 General bottom-up proteomics approaches for studying PTMs

MS-based proteomics can be broadly divided into bottom-up and top-down approaches, and both methods have been used to study host responses to viral infection (78). Decades of advancements in sample preparation, MS instrumentation, and data analysis of bottom-up proteomics enable in-depth profiling of the PTM landscape in a biological system. A brief primer on bottom-up proteomics for PTM profiling is provided in this section and interested readers are directed to other review literature for a thorough update (79–83). Applications employing the bottom-up approach in studying host-virus interactions will be discussed in Sections 5 and 6.

**Figure 4:**
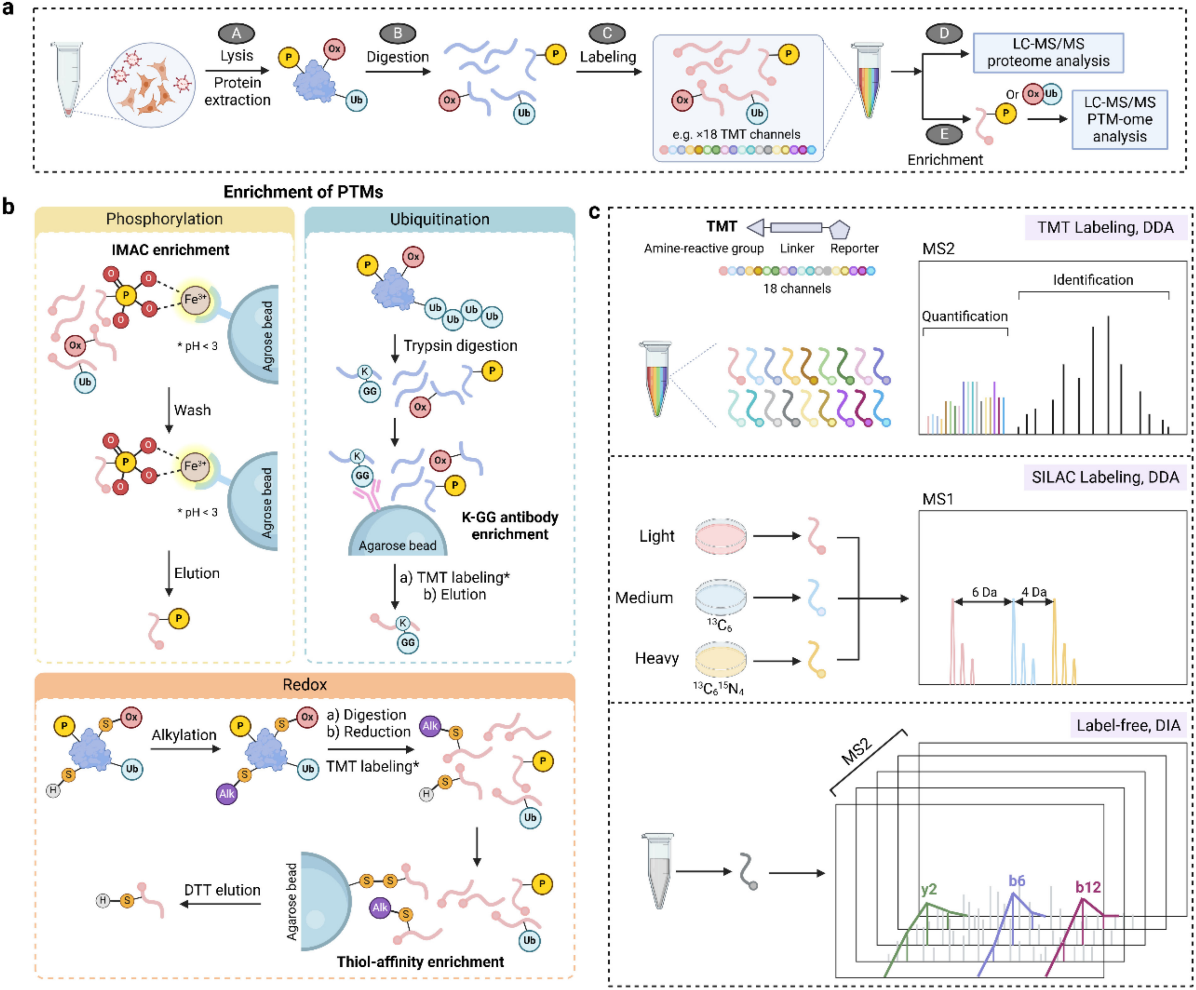
MS-based proteomics approaches for studying PTMs. **(a)** A general workflow for PTM proteomics consisting of cell/tissue lysis, protein extraction and digestion, optional labeling, enrichment, and LC-MS/MS analysis. **(b)** Enrichment strategies for phosphorylation (IMAC), ubiquitination (affinity enrichment via K-GG antibody), and reversible thiol oxidation (thiol affinity enrichment). **(c)** Quantification approaches in PTM proteomics including isobaric labeling (TMT), SILAC, and label-free methods. DIA, data independent acquisition; DTT, dithiothreitol; IMAC, immobilized metal ion affinity chromatography; MS1, mass spectra of peptide precursors; MS2, mass spectra of peptide fragments; TMT, tandem mass tags. * in panel b denotes TMT labeling is optional.

In general, bottom-up proteomics approaches for PTM characterization consist of protein extraction, trypsin digestion, optional labeling of the resulting peptides, enrichment for PTMs of interest, and finally LC-MS/MS (**Figure 4a**). One of the most critical considerations in PTM study design is to preserve the endogenous modified residues due to their labile nature and low abundance under physiological conditions. This requires specific quenching/stabilization and enrichment techniques to enable sufficient coverage of the PTM proteome. In a phosphoproteomics experiment, phosphatase inhibitors are routinely included in sample harvesting and protein extraction buffers to avoid losing *in situ* phosphorylation. Similarly, suberoylanilide hydroxamic acid and nicotinamide are typically added to the lysate buffer to inhibit the activity of deacetylases (84). When performing redox proteomics experiments, gentle lysis conditions are preferred to reduce sample exposure to artifactual oxidation. Alkylation reagents (e.g., N-ethylmaleimide or iodoacetamide) are usually employed to block reactive cysteine thiols to minimize false-positive identification and disulfide scrambling. Acidic conditions such as trichloroacetic acid incubation is an alternative way to stabilize redox proteome.

PTMs can be captured non-covalently or covalently through a variety of mechanisms (**Figure 4b**). Taking advantage of the non-covalent interaction between negatively charged phosphate groups and positively charged metal ions, immobilized metal ion affinity chromatography (IMAC) and metal oxide affinity chromatography (MOAC) are the most widely applied enrichment methods for phosphorylation (85). In such approaches, pH is the most important factor for enrichment selectivity because it should promote the deprotonation of the phosphate group (thus a negative charge) and protonation of acidic amino acids (aspartate and glutamate residues). Enrichment of lysine ubiquitination exploits the non-covalent interaction between K-ϵ-GG antibody and the characteristic diglycine remnant after trypsin digestion of ubiquitin (86, 87). A similar antibody-based enrichment is widely employed for lysine acetylation (88), where high-quality antibodies are commercially available (e.g., anti-Acetyl-Lysine immunoaffinity magnetic bead). In addition to the non-covalent approaches, the functional group of a PTM can form a covalent bond with the capturing reagent and thus be enriched. One example is the resin-assisted capture, in which the protein thiol group is attached to a Thiopropyl Sepharose 6B affinity resin via thiol-disulfide exchange (89). In general, PTM enrichment is technically challenging and laborious. To improve the throughput and reproducibility, much effort has been spent on automation of these approaches including the use of magnetic bead-based systems.

Quantification of PTMs can be achieved using isotope labeling (e.g., stable isotope labeling by amino acids in cell culture, SILAC), isobaric labeling (e.g. tandem mass tags, TMT), and label-free approaches (**Figure 4c**). SILAC is performed by including light or heavy (e.g., ^13^C, ^15^N) isotope-labeled amino acids in cell culture medium to metabolically label proteins, yielding a mass shift of precursor peaks in MS1 for relative quantification. While SILAC is generally limited to cell culture experiments, isobaric labeling of peptides is compatible with a wide range of sample types and offers greater multiplexing and throughput. For instance, TMT reagents permit the multiplexed quantification of up to 35 samples in one LC-MS run. TMT tags react with primary amine groups of peptides and the intensity of the reporter groups is used for relative quantification. Because of a small mass difference among TMT reporter groups (0.0063 Da), a high resolution Orbitrap is typically used to resolve the mass spectra. Similar to TMT, the amine-reactive N,N-dimethylleucine (DiLeu) isobaric tags can multiplex up to 21 samples with one set of reagents, which can be synthesized in the lab and provides a cost-effective alternative (90). Moreover, a hyperplexing strategy that combines triplex SILAC labeling and six-plex isobaric labeling has also been reported (91). Although isobaric labeling offers increased multiplexity and is a mainstay for quantitative proteomics, it can also suffer from ratio compression due to co-eluted and co-fragmentated peptides. This can be partially alleviated by a first-dimensional fractionation of the peptide samples or performing quantification at the MS3 level (92).

Recently, data independent acquisition (DIA) emerged as a powerful tool to quantify protein PTMs via a label-free strategy. Reproducible and site-specific quantification of ~ 35,000 phospho-sites and diGly sites was achieved in a single measurement with constructed hybrid phosphoproteome and ubiquitinome spectral libraries, respectively (93, 94). Direct DIA without the need of spectral libraries also allowed quantifying over 20,000 phosphopeptides in a single-shot analysis using a short gradient (15 min) (95). However, DIA has significant challenges due to the increased spectral complexity including false discovery rate (FDR) control for PTM identification and the accuracy of PTM site localization.

## 4 Top-down approaches for studying combinatorial PTMs

While bottom-up workflows entail the enzymatic digestion of proteins into peptides prior to MS analysis, top-down workflows directly characterize intact proteins. Therefore, the top-down approach is advantageous in preserving information regarding combinatorial PTMs (96). Top-down proteomics can be further divided into denatured and native workflows, with native experiments using buffered solutions to maintain protein folding and non-covalent interactions between protein complexes, co-factors, ligands, etc. While bottom-up proteomics is technically mature and has been routinely used, significant technical advancements in top-down proteomics are still needed to characterize PTMs, including separation of proteoforms bearing different PTMs, detection of large proteins (> 30 kDa), and fast and accurate proteoform identification with an increasingly large number of theoretical combinations of PTMs.

A unique strength of top-down approaches is that they can reveal the degree of heterogeneity and combinatorial PTMs. Histones are a prime example of proteins with multiple, co-occurring PTMs integrated into unique gene expression patterns, dubbed the “histone code” (97, 98). These PTMs regulate chromatin structure, recruitment of expression factors, and ultimately gene expression levels. In a recent study, top-down analysis of intact histone fractions derived from T cells pre- and post-activation following influenza infection showed many combinatorial modifications that are unique to either the naïve or activated states (99). Notably, naïve T cells presented more known enhancers of gene expression. However, the application of top-down proteomics for histone modifications in the context of viral infection is still limited, and more case studies are expected to reveal the true landscape of histone code under viral infection.

Viral capsid proteins are highly glycosylated, with glycans mediating many aspects of viral pathobiology including host-cell entry and immune evasion (1, 100). Several advancements in top-down workflows, including high-resolution mass analyzers (101) and single-particle MS techniques (102–104), have allowed for direct visualization of intact glycoforms of either individual or partially assembled capsid proteins. Native MS has also provided unique insights regarding the assembly process and protomer stoichiometry of viral protein complexes (78, 105), however it is currently unclear how PTMs of viral proteins may regulate these processes.

Another emerging field is the use of top-down approaches to characterize the PTM landscape of antibodies from serum/blood samples (106). Host antibodies are subject to glycosylation that controls antibody specificity (105, 107). Top-down analysis of antibody repertoires offers information regarding total glycosylation status as well as chain pairing and disulfide connectivity (108). However, challenges in separation, limited sequence coverage during MS/MS, and the high degree of sequence and modification heterogeneity in antibodies limit top-down applications of complex, serum-derived samples (109). In addition, single particle MS measurements have also provided new insights into antibody repertoires (110), including a recent example of characterizing antibody repertoires against the receptor binding domain (RBD) of SARS-CoV-2 in unvaccinated, vaccinated, and infected SARS-CoV-2 patients (111). Following dimensionality reduction of the intact antibody data, the top-down approach differentiated uninfected versus hospitalized or vaccinated individuals.

## 5 PTM-omics insights into host cellular response to viral infection

**Figure 5:**
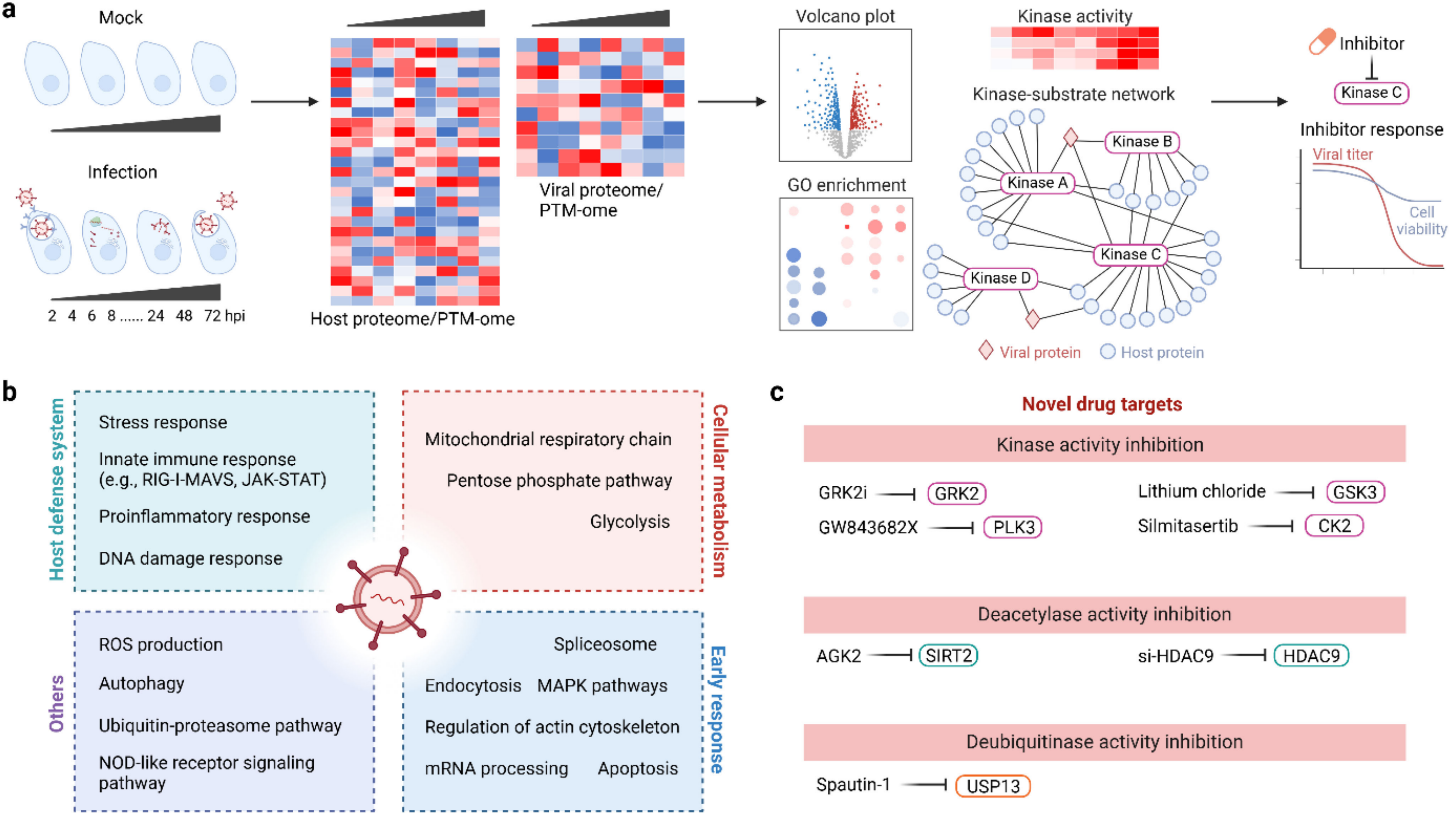
Use PTMomics to understand host responses to viral infection and to develop novel antiviral therapies. **(a)** A typical experimental setup and data analysis pipeline for PTMomics studies. **(b)** Common cellular pathways that respond to viral infection. **(c)** Novel antiviral drugs that target protein PTMs. CK2, casein kinase II; HDAC9, histone deacetylase 9; GRK2, G protein-coupled receptor kinase 2; GSK3, glycogen synthase kinase-3; PLK3, polo-like kinase 3; SIRT2, sirtuin 2; USP13, ubiquitin-specific protease 13.

A major motivation of PTM-omics studies in infected cells is to better understand the host response to viral infection at the molecular level (**Figure 5**). PTMs are dynamic, and time course PTM profiling can reveal a temporal PTM landscape and how PTM dynamics lead to shifting of cellular functions to enhance viral replication. Previous PTM time course studies have demonstrated that virus infection causes extensive changes in the host protein PTM landscape, impacting multiple cell signaling pathways including metabolism, intracellular trafficking and transport, and innate immune responses during viral infection. Equally important, rich PTM data can be used to identify potential biomarkers and drug targets from thousands of quantified features, accelerating the screening and rationale design for therapies against infectious diseases. In this section, we will showcase selected studies that employ PTM-omics techniques to understand PTM-mediated cellular pathways in viral infection and to develop novel anti-viral therapies based on the PTM mechanisms of host cellular proteins. A subset of lead compounds from these studies that exploit PTM mechanisms for antiviral treatment are summarized in **Table 1**.

**Table 1:** Selected compounds exploiting PTM mechanisms for antiviral treatment.

Compound	Virus	Mode of action (MOA)	Ref
Silmitasertib	SARS-CoV-2	Inhibition of CK2, which has physical interaction and co-localization with viral N protein	(19)
Gilteritinib, ralimetinib, MAPK13-IN-1 and ARRY-797	SARS-CoV-2	Inhibition of the upstream driver (AXL) and members (MAPK11/13/14) of p38 signaling pathway	(19)
Apilimod	SARS-CoV-2	Inhibition of PIKFYVE to regulate phosphatidylinositol species	(19)
Dinaciclib	SARS-CoV-2	Inhibition of CDK signaling to interrupt viral regulation of cell cycle to enhance viral replication	(19)
Sapanisertib, samotolisib, and gedatolisib	SARS-CoV-2 and MERS-CoV	Inhibition of PI3K/mTOR signaling pathway	(113)
AGK2	HCMV	Inhibition of SIRT2 deacetylase activity and alters CDK2 acetylation and cell cycle	(119)
Lithium chloride	SARS-CoV-2	Inhibition of GSK3 activity to block viral N protein phosphorylation and viral replication	(132)
Spautin-1	SARS-CoV-2	Inhibition of USP13 deubiquitination activity which leads to nsp13 degradation and promoting type I IFN production	(139)
Aminoadamantane nitrate compounds (e.g., NMT5)	SARS-CoV-2	Mediating S-nitrosylation of ACE2 to inhibit spike protein binding to ACE2 and viral entry into host cells	(151)
FLS-359	RNA and DNA viruses*	Inhibition of SIRT2 deacetylase activity	(154)
PROTACs (e.g., BP-198)	SARS-CoV-2	Binding M^pro^ and recruiting ubiquitin ligase machinery to degrade M^pro^	(155)

ACE2, angiotensin converting enzyme 2; CDK2, cyclin-dependent kinase 2; CK2, casein kinase II; GSK3, glycogen synthase kinase-3; MAPK11/13/14, mitogen-activated protein kinase 11/13/14; MERS-CoV, Middle East respiratory syndrome coronavirus; HCMV, human cytomegalovirus; PI3K, phosphatidylinositol 3-kinase; PIKFYVE, 1-phosphatidylinositol 3-phosphate 5-kinase; SARS-CoV-2, severe acute respiratory syndrome coronavirus 2; SIRT2, Sirtuin 2; USP13, deubiquitinating enzyme 13.

*e.g., coronaviruses, flaviviruses, and herpesviruses.

### 5.1 Phosphorylation

Viral infections can induce host cell protein phosphorylation which can happen within minutes of infection. Yángüez et al. conducted a SILAC-based quantitative phosphoproteomic analysis to capture the rapid and systematic response induced by IAV in human lung epithelial cells (112). Triple isotope labeling was performed by adding labeled amino acids to the culture media and samples were taken at 5 and 15 minutes post-infection and compared to mock-infected cultures. These studies revealed early phosphorylation signatures including MAPK signaling, endocytosis and regulation of the actin cytoskeleton upon viral entry (**Table 2**). The authors used pathway enrichment analysis of the differentially phosphorylated proteins and prediction analysis to identify protein kinases responsible for the phosphorylation dynamics leading to the identification of G protein coupled receptor kinase 2 (GRK2) as a host factor required for IAV uncoating.

**Table 2:** Selected recent MS-based proteomic studies of PTMs in virus infection.

PTM	Virus	Host	Time points	Samples	Enrich	Quan	PTMome Coverage	Major Findings	Ref
Phos	HBV	Primary human hepatocytes	2/7 dpi	12	TiO_2_	TMT	8,308 P peptides; 3,012 proteins	Upregulation of DNA repair as a major anti-viral response	(156)
Phos	HIV-1	Jurkat E6.1 T cells	24 hpi	15	IMAC	LF/ DIA	14,105 P sites; 6,078 proteins	HIV-1 requires Aurora kinase activity for replication in human T cells and macrophages	(4)
Phos	IAV	A549 cells	5/15min	15	IMAC/ TiO_2_	SILAC	2400 P peptides; 1300 proteins	Phosphorylation changes were observed in minutes upon infection. G protein-coupled receptor kinase 2 (GRK2) were activated by viral infection. Inhibition of GRK2 impaired IAV uncoating and viral replication.	(112)
Phos	DENV	K562 cells	48 hpi	6	TiO_2_	Dimethyl	2440 P sites; 2263 proteins	Phosphoproteins were involved in transcription regulation, RNA splicing and processing, immune system, cellular response to stimulus, and macromolecule biosynthesis in response to Dengue virus infection.	(157)
Phos	SARS-CoV-2	Vero E6 cells	2/4/8/12/24 hpi	24	IMAC	DIA	4,624 P sites; 3,036 proteins	SARS-CoV-2 infection promoted activation of p38 MAPK and CK2, cytokine production, shutdown of mitotic kinases, leading to cell cycle arrest.	(19)
Phos	SARS-CoV-2/ SARS-CoV	A549-ACE2 cells	6/12/24/36 hpi	48	IMAC	LF/ DIA	16,399 P sites	Highly regulated sites were identified in MAPK pathway, autophagy signaling, viral entry. Differential activation of EIF2AK2 during infection by two viruses may contribute to different growth kinetics of the two viruses. Interplay between Phosphorylation and Ubiquitination can be found on individual host protein (e.g., EGFR).	(123)
Ubi	HIV-1	Jurkat E6.1 T cells	24 hpi	32	IP	SILAC	11,821 U sites	HIV-1 Vpr expression reduced histone H1.2 ubiquitination via CRL4^DCAF1^	(4)
Ubi	SARS-CoV-2/ SARS-CoV	A549-ACE2 cells	6/12/24 hpi	36	IP	LF/ DIA	16,541 U sites	Shared and distinct ubiquitination signatures were observed during infection by two viruses. Several proteins exhibit ubiquitination-mediated protein degradation.	(123)
Ubi	SARS-CoV-2	Vero E6 cells	72 hpi	6	IP	LF	8943 U sites; 3086 proteins	SARS-CoV-2 may inhibit IFN-I signaling and antiviral response by modulating ubiquitination level of USP5	(115)
Acetyl	Human cytomegalovirus (HCMV)	MRC5 human lung fibroblasts	24/48/72/96 hpi	15	IP	LF	6180 A sites; 2018 proteins	Temporal and spatial acetylome across infection time. Lamin B1^K134^ acetylation at the nuclear periphery inhibited capsid nuclear egress and virus production.	(118)
FAT10	KSHV	iSLK-rKSHV.219 cells	12 hpi	4	IP	LF	1,402 proteins	KSHV ORF59 and ORF61 were FAT10ylation substrates	(140)

Acetyl/A, acetylation; HBV, hepatitis B virus; dpi, days post infection; DENV, Dengue virus DIA, data-independent acquisition; HCMV, Human cytomegalovirus; KSHV, Kaposi’s sarcoma-associated herpesvirus; hpi, hours post infection; IAV, influenza A virus; IP, immunopurification; Phos/P, phosphorylation; TMT, tandem mass tags; Ubi/U, ubiquitination.

In another study, Bouhaddou et al. quantified the phosphorylation landscape of Vero E6 cells following SARS-CoV-2 infection at six time points (0, 2, 4, 8, 12, or 24 h post-infection) (19). Using DIA for MS data collection and label-free quantification, 4,624 phosphorylation sites (3,036 proteins) were found to show temporal dynamics following infection. Based on the change in the phosphorylation level of substrate proteins, the authors estimated the kinase activity of 97 kinases and found that viral infection strongly activates many kinases including CK2 and p38 MAPK. Accordingly, the antiviral efficacy of 68 kinase inhibitors targeting the most active kinases were evaluated in both Vero E6 and A549-ACE2 cell lines. Inhibitors targeting CK2, pp38 MAPK signaling, PIKFYVE and CDKs were efficacious against SARS-CoV 2. Of note, CK2 inhibitor Silmitasertib is currently in clinical development for treating patients with COVID-19. Besides discovering kinases that can regulate virus replication by prediction based on phosphorylation of known substrates, host kinome responses can also be probed using multiplex inhibitory bead mass spectrometry (MIB-MS) as demonstrated by Fritch et al. in human lung epithelial cells infected by MERS-CoV and SARS-CoV-2 (113). With paired phosphoproteomics and chemogenomic screening, PI3K/mTOR inhibitors (sapanisertib, samotolisib, and gedatolisib) showed potent antiviral activity for both MERS-CoV and SARS-CoV-2.

Phosphoproteomics studies can also be utilized on clinical samples to understand the molecular basis of symptoms in infectious disease patients. In a recent study, Kaneko et al. characterized the dynamic proteome and phosphoproteome in septic patients with and without COVID-19 (n = 5 for each group, along with 5 healthy controls) (114). They collected patient blood samples 1, 7, and 10 days after COVID-19 infection and isolated peripheral blood mononuclear cells (PBMC) for proteome and phosphoproteome measurements. They quantified ~2400 Ser/Thr/Tyr phosphorylation sites including 380 unique pTyr sites. The unusually high percentage of pTyr sites (< 1% in most phosphoproteomics studies) is due to the use of a boosting TMT channel for Tyr phosphorylation, in which PBMCs were treated with a pervanadate solution. Samples from COVID-19 patients had significant changes in the phosphoproteome and kinome when compared to healthy controls. CK2 was identified again in this study by kinase-substrate enrichment analysis (KSEA) emphasizing its therapeutic potential. Interestingly, a significant difference in Tyr phosphorylation on immune regulators was found comparing COVID-19 patients to healthy controls, highlighting the role of Tyr phosphorylation in mediating the signaling pathway of immunoreceptors and cytokines, which suggested patients possessed partially activated T cells despite having lymphopenia, as well as compromised myeloid and NK cell effector functions.

### 5.2 Ubiquitination

By applying label-free and antibody-based quantitative ubiquitinomics analysis, Zhang et al. identified 8,943 lysine ubiquitination sites in SARS-CoV-2 infected Vero E6 cells (at 72 h post-infection) and identified 966 differentially modified sites on 551 proteins (115). The differentially ubiquitinated proteins were enriched in both metabolism pathways (e.g., pentose phosphate pathway) and signaling pathways (e.g., NOD-like receptor signaling pathway). Although the biological relevance of these ubiquitination events remains unknown, many differentially ubiquitinated proteins are key modulators of host immune responses. For instance, NOD-like receptor family pyrin domain containing 3 (NLRP3) is a key component of the inflammasome and when activated causes the release of proinflammatory cytokines, contributing to virus-induced inflammatory cytokine storm and more severe disease outcomes.

To gain a better understanding of the pathogenesis of SARS-CoV-2, Xu et al. profiled the ubiquitinome, along with the transcriptome and proteome, of human lung epithelial Calu3 cells infected with SARS-CoV-2 (116). At 24 h post-infection, a great shift in the number of ubiquitinated lysine sites was observed with 5,359 ubiquitinated lysine sites on 2,124 proteins up-regulated and 1,176 ubiquitinated lysine sites on 675 proteins down-regulated. Similar to Zhang et al. (115), the authors found a significant change in lysine ubiquitination on proteins involved in the host innate immune signaling pathways including RIG-I-MAVS and JAK-STAT pathways. Interestingly, the multi-omics analysis enabled the identification of host antiviral proteins with increased RNA levels but decreased protein abundances due to significantly changed ubiquitination levels, indicating SARS-CoV-2 likely hijacked the ubiquitin-proteasome system to facilitate viral replication. Potential mechanisms included interaction of the nonstructural protein 3 (nsp3) SARS-unique domain (SUD) and/or papain-like protease (PL^pro^) with and stabilization of host E3 ubiquitin ligase RING finger and CHY zinc-finger domain containing protein 1 (RCHY1) thus promoting RCHY1-mediated p53 degradation (117).

### 5.3 Acetylation

Acetylation dynamically modulates protein functions in host cells across different stages of the viral replication cycle. To elucidate the regulatory role of protein acetylation during human cytomegalovirus (HCMV) infection, Murray et al. compared the acetylome in infected and mock MRC5 cells at various stages of viral infection (24, 48, 72, and 96 hours post-infection) (118). They identified 6,180 acetylated peptides (on 2,018 proteins) and observed temporal regulation of acetylation. Integrating the acetylation data with subcellular location and protein abundance, the authors suggested that acetylation of Lamin B1^K134^ impedes the disruption of the nuclear lamina and thus the egress of viral capsids from the nucleus and viral production. Modulating acetylation levels during viral infection could be a potential therapeutic strategy, which necessitates a better understanding of enzymes that regulate cellular acetylation.

In a follow-up study, the same group discovered that sirtuin 2 (SIRT2), an enzyme with deacetylase activity, regulates the acetylation of cellular proteins including cell cycle proteins during infection and its activation promotes HCMV replication (119). Because SIRT2 has various enzymatic activities beyond deacetylation (e.g., defatty-acylase function (120)), the authors treated cells with AGK2, a SIRT2 inhibitor that specifically inhibits the deacetylase activity. They found that inhibition of SIRT2 deacetylase activity alters the acetylation CDK2^K6^, which drives the G1/S transition to prevent HCMV replication. Thus, the SIRT2-CDK2 axis represents a novel target for developing anti-viral drugs. A similar acetylation-dependent regulation has also been reported in Caco-2 cells infected with rotavirus (RV), in which histone deacetylase 9 (HDAC9) promotes the deacetylation of glyceraldehyde-3-phosphate dehydrogenase (GAPDH^K219^) and RV replication (121). However, it remains unclear whether such regulatory axes are specific to a virus strain or a host cell line.

Acetylome profiling has also been conducted in clinical samples to discover potential prognostic markers. Chai et al. quantified 2,492 acetylation sites of 1,190 proteins in normal, paracancerous, and hepatocellular carcinoma (HCC) liver tissues from hepatitis B virus (HBV)-infected patients (122). In addition, acetylated proteins were enriched in viral carcinogenesis and HBV pathways. Interestingly, a small group of proteins were found multi-acetylated on more than 10 lysine residues. Among them, the top three proteins are mitochondrial 60 kDa heat shock protein (HSPD1), trifunctional enzyme subunit alpha (HADHA), and carbamoyl-phosphate synthase (CPS1), which may be involved in the response to HBV-associated liver cancer progression. Furthermore, hyperacetylated histones including H2B^K120^, H3.3^K18,^ and H4^K77^ were detected in HCC samples and were associated with a worse disease outcome prognosis (i.e., reduced survival and higher recurrence) in an independent cohort of 135 HCC patients. Thus, these histone acetylation sites can serve as prognostic biomarkers.

### 5.4 Multi-PTM profiling of cellular responses to viral infection

The presence of multiple PTM simultaneously on the same protein allows interaction or crosstalk among them, which together determine the function of a protein. Characterization of multi-PTM types within a single sample is thus of great value to reveal PTM crosstalk and the cell signaling mechanisms that combinatorial PTMs may regulate. Proteome-wide assessment of multi-PTM types from the same sample set, however, has been limited by the lack of integrated experimental tools. Instead, a common approach in multi-PTM studies involves the preparation of a separate sample set for each PTM type of interest. For example, Johnson et al. prepared two sets of Jurkat E6.1 T cells (infected with HIV-1 for 24 h) and quantified the changes in phosphorylation and ubiquitination, respectively, to better understand virus host interactions (4). To examine phosphorylated proteins in response to HIV infection, the authors used Fe^3+^-immobilized IMAC for enrichment and a label-free DIA approach for quantification. Unenriched samples were used to quantify the protein abundance. To examine proteins that were ubiquitinated in response to HIV infection, mock-infected and HIV-infected cells were cultured in different SILAC media and were treated with a proteasome inhibitor (MG-132) to preserve ubiquitination targets before sample harvest. Enrichment was performed using the ubiquitin remnant motif (K-ϵ-GG) immunoaffinity beads. In total, 11,821 ubiquitination sites, 14,105 phosphorylation sites, and 6,078 proteins were quantified. Using the phosphorylation data, the authors predicted the kinases most likely to be active during HIV-1 infection and found that inhibition of the Aurora kinase impaired HIV-1 replication suggesting it is important for productive infection. This study demonstrated that the HIV-1 accessory protein Vif1-mediated ubiquitination and degradation of the B56 family of protein phosphatase 2A regulatory subunits (PP2A-B56), which is involved in cell-cycle arrest. Analysis of the phosphoprotoemics data revealed putative PP2A-B56 substrates including Rho guanine nucleotide exchange factor 2 (ARHG2) supporting crosstalk between phosphorylation and ubiquitination signaling, in which the viral protein Vif1 modulates the ubiquitination of host protein PP2A-B56, which in turn regulates the activity of ARHG2 by phosphorylation.

In another multi-PTM study, A549 cells were infected with SARS-CoV-2 or SARS-CoV, and samples were harvested at 6, 12, and 24 h post-infection (123). For multiomics analysis, three aliquots for each sample were taken for proteome, phosphoproteome, and ubiquitinome measurement via a DIA approach. As expected, a large number of proteins bearing both phosphorylation and ubiquitination were found, especially proteins involved in viral entry. This integrated PTM analysis underscores the critical role of combined PTMs in fine-tuning host cellular responses to viral infection, ultimately impacting signal transduction, protein stability and immune evasion mechanisms. However, the spitting of samples for multiomics and DIA for data acquisition creates many LC-MS runs (three times of the sample number), which can be mitigated by either sample multiplexing or shorter LC-MS runs. While phosphorylation and ubiquitination are on different amino acids, other types of PTMs can occur on the same residues and are multi-exclusive. Ahmed et al. probed acetylation, methylation, and allysine modification (a type of oxidative deamination of lysine) in A549 infected with IVA (124). They found many cases of co-modification with two or more PTMs on the same residues, including methylation and acetylation of a subunit of the IAV RNA polymerase (PA^K102/K014^). While this study reveals the dynamic and combinatorial natures of modifications, the biological significance of the complicated PTM patterns (e.g., how multiple PTMs are integrated into one signal as in the case of “histone code”) remains elusive.

## 6 PTMs of viral proteins revealed by proteomics

PTM modification of viral proteins has been long recognized as critical for virus replication (125, 126). Viruses are minimalists and their genomes only encode required functions and can range in size from 1 to 2 (smallest) to 1,300 (largest) kilobases. Thus, many viral proteins are multifunctional, which can be mediated by various PTM events that endow new/altered functions during the infection cycle (127). Although some viruses encode PTM writers such as protein kinases (128) and acetyltransferases (129), most viruses rely on the host cellular PTM machinery (e.g., protein kinases, E3 ubiquitin ligases, and lysine acetyltransferases for phosphorylation, ubiquitination, and acetylation, respectively) to add PTMs to viral proteins (**Table 3**). Identifying the interaction between host PTM writers/erasers and viral proteins is an area of growing research interest, and targeting these cellular regulatory proteins (e.g., by pharmacological inhibition) presents a viable and effective response for developing anti-viral therapies. As proteomics studies can reveal novel PTM sites on viral proteins, studying the functional impact of these modifications should shed light on how PTMs regulate various aspects of virus replication and how this knowledge can be used to develop therapeutic strategies (**Table 3**).

**Table 3:** Selected viral proteins with PTMs and their functional roles.

Viral proteins*	Host enzymes	Biological function	Ref.
RV dNSP2-S313	CK1α	Phosphorylation triggers localization of vNSP2 to viroplasm assembly sites	(158, 159)
SARS-CoV-2 N-S188, S206	GSK-3	Phosphorylation important for intracellular viral RNA accumulation and viral replication	(132)
HCV core protein-R9, K12, R81, S99, R101, S106, R117, and S145	TAB1-p38	Phosphorylation important for oligomerization of HCV core protein and HCV assembly	(160)
H1N1 NS1-S205	CK2	Phosphorylation required for interaction with DDX21	(135)
HPV16 L2-T214	CDK1 and PLK1	Phosphorylated during mitosis and dephosphorylated in G1/S-phase) to prime binding and sequential phosphorylation (T265) by PLK1	(161)
EBOV VP30-S29	SRPK1	Phosphorylation enabled viral transcription	(162)
IBDV VP3-S13	Cell division cycle 7-related protein kinase	Phosphorylation regulated viral replication	(163)
H1N1 nucleoprotein-S482	PLK3	Phosphorylation facilitate NP oligomerization, vRNP assembly, viral polymerase activity	(136)
IAV polymerase PB1-K578	–	Ubiquitination disrupts interaction between PB1 thumb domain and PB2 N-terminus, thus mediating polymerase dimerization and vRNA replication	(138)
IAV PB2-K29	Cullin 4-based multicomponent RING-E3 ligases	Ubiquitination during infection via interaction with CRL4 factors	(127)
SARS-CoV-2 nsp13	USP13	De-ubiquitination via interacting with USP13 to remain protein level	(139)
SARS-CoV-2 S-K310, K986, and K1028	–	Ubiquitination affects cleavage of S into S1 and S2 subunits by furin and TMPRSS2, thus reduces viral fusion with the cell membrane	(116)

*The PTM site is given if available, in the format of single letter amino acid denotation followed by location of PTM in protein sequence. CDC7, cell division cycle 7-related protein kinase; CDK1, cyclin-dependent kinase 1; CK1a: casein kinase Ia; CK2, casein kinase II; EBOV, Ebola virus; GSK3, glycogen synthase kinase-3; H1N1, Influenza A virus subtype H1N1; HCV, Hepatitis C Virus; IAV, influenza A virus; IBDV, Infectious bursal disease virus; PLK1, polo-like kinase 1; PLK3, polo-like kinase 3; RV, rotavirus; SARS-CoV-2, severe acute respiratory syndrome coronavirus 2; SRPK1, serine-arginine protein kinase 1; TAB1, TGF-beta activated kinase 1 (MAP3K7) binding protein 1; USP13, deubiquitinating enzyme 13.

### 6.1 Phosphorylation

Phosphorylation is one of the most abundant PTM of viral proteins. Since the majority of viral genomes do not encode protein kinases (130), viruses exploit the host PTM machinery to modify their proteins to facilitate viral entry, replication, assembly, and evading host immune responses (126). For example, the SARS-CoV-2 nucleocapsid (N) has been reported to be phosphorylated by the host protein glycogen synthase kinase-3 (GSK3) (19, 131, 132). Phosphorylated N protein from the JHM strain of mouse hepatitis virus (MHV strain JHM) recruits the RNA helicase DDX1, which is required for the replication of some RNA viruses (133, 134). GSK3 seems to be the primary kinase for the N protein, whose phosphorylation is impaired by either genetically knocking out the GSK3 genes (*GSK3A* and *GSK3B*) or chemically inhibiting GSK3 activity (e.g., lithium chloride) (132). When N phosphorylation was inhibited, significantly reduced intracellular viral RNA levels and viral titers were observed. This qualifies selective GSK3 inhibitors as potential antiviral candidates.

Phosphoproteomics studies have revealed that IAV nonstructural protein 1 (NS1), a key player in viral replication, is phosphorylated at S205 and NS1’s phosphorylation represents a mechanism by which viruses use PTMs to enhance viral polymerase activity for genome replication (135). By generating virus mutants harboring NS1^S205G^ that cannot be phosphorylated and NS1^S205D^ (phosphomimetic), Patil et. al., found that phosphorylation of NS1^S205^ is required for binding with the cellular interaction factor DDX21 to promote viral polymerase activity. Phosphorylation of NS1^S205^ is catalyzed by the cellular kinase CK2, and inhibition of CK2 activity leads to decreased NS1–DDX21 interaction and reduced IAV replication. Host kinase-mediated phosphorylation was also found to directly impact polymerase activity by modifying IAV H1N1 nucleoprotein (NP) (136). The authors demonstrated that host polo-like kinase 3 (PLK3) phosphorylated NP^S482^ which enhanced the assembly of the viral ribonucleoprotein complex (which includes NP, viral RNA, and subunits of polymerase) and viral polymerase activity.

### 6.2 Ubiquitination

Viruses can also exploit the host cellular ubiquitination system to benefit multiple aspects of their replication cycle, from entry and replication to immune evasion and virus assembly (137). However, the functional impact of ubiquitination on viral proteins is less understood than that of phosphorylation. In a recent seminal study, Günl et al. infected A549 cells with human IAV A/WSN/1933 and used MS-based proteomics to map the ubiquitination landscape of the IAV RNA-dependent RNA polymerase (138). They identified 59 well-conserved modified lysine residues on the three subunits of the IAV polymerase (PB2, PB1, and PA), and most are located on the polymerase trimer surface. Subsequent functional studies revealed that ubiquitination on PB1^K578^ causes structural transitions of the viral polymerase, possibly by neutralizing the positive charge of the lysine residue and thus changing the binding pattern of PB1. Thus, this study demonstrates that ubiquitin-mediated charge neutralization can act as a switch to tune protein activities and that maintaining the charge status of key lysine residues is vital for a polymerase complex’s timely transition in various functional states during the virus replication cycle.

As most viral genomes do not encode E3 ligases, most viruses rely on the cellular ubiquitination system to catalyze the ubiquitination of proteins during infection making these enzymes candidates for host-based antivirals. Ubiquitination of SARS-CoV-2 spike glycoprotein at K310, K986, and K1028 enhances viral infection in lung epithelial Calu3 cells and site-directed mutagenesis at these sites leads to significantly reduced infection (116). To develop a ubiquitination-based antiviral therapy, Zhang et. al., screened a library of ubiquitination enzymes and found that overexpressing twelve E3 ubiquitin ligases and four deubiquitinating enzymes alters the level of SARS-CoV-2 viral load. These results were validated by knocking down the expression of these enzymes and demonstrated that manipulating the host ubiquitination system is a viable way to reduce viral replication. Other examples include two E3 ubiquitin ligases called RING-E3 ligases based on cullin 4 (CRL4), which catalyze nonproteolytic K29-linked ubiquitination of IAV PB2 protein to mediate viral replication cycle progression and virion production (127). In addition, deubiquitination enzymes can also be hijacked by viruses to maintain their protein stability and suppress the host’s immune response. For example, SARS-CoV-2 non-structural protein 13 (nsp13) was reported to interact with host deubiquitinase ubiquitin-specific protease 13 (USP13) to remove ubiquitin moieties and stabilize itself (139). Thus, USP13 knock-down or inhibition led to increased ubiquitination of nsp13 and its degradation which reduced nsp13 inhibitory effect on IFN-β production.

## 7 Concluding remarks and perspectives

Diverse PTMs fine-tune protein functions and play a critical role in immune responses. PTM writers and erasers are also promising anti-viral targets as viruses hijack the cellular PTM machinery and turn a host cell into a virus factory. PTMs are highly dynamic and prevalent in host defense response and the entire viral replication cycle, highlighting the need for quantitative and systematic characterization of PTMs to comprehensively understand virus-host interactions. Indeed, the last decade has seen the upsurge of MS-based PTM proteomics studies of infectious disease models, revealing widespread PTM events that are involved in either pro- or anti-viral mechanisms. While phosphorylation, ubiquitination, and acetylation have been frequently studied, other PTMs such as the addition of ubiquitin-like protein (FAT10) to lysine residues that regulate the inflammatory response pathway are less well understood (140). Moreover, many physiologically important PTMs in the context of viral infection such as thiol oxidation have not been explored at the proteome-level. A better understanding of how PTM contribute to host-virus interactions would also inspire the development of new anti-viral interventions. Future PTM studies in primary cells or patient-derived biofluid (e.g. PBMCs) are anticipated to provide more physiologically relevant model systems to examine PTM landscape dynamics during viral infection. Such studies could reveal how PTMs contribute to the heterogeneous molecular responses between individuals to viruses and provide PTM measurements to assess the overall fitness of the immune system. Despite its potential, PTM analysis of biofluid samples remains largely underexplored due to technical challenges such as sample instability and high molecular complexity. Overcoming these hurdles—along with innovations that reduce the cost of PTM analysis—will enhance the feasibility of applying PTMomics to biofluid samples in clinical settings.

With advances in sample processing and cutting-edge instrument development, the field of PTM proteomics is constantly evolving with significant improvements in proteome coverage, reproducibility, multiple-PTM integration, and throughput. For instance, the first acetylome study of HCMV-infected samples a few years ago identified a few thousand acetylated peptides (118), possibly due to a low enrichment selectivity (~30% of peptides are acetylated) of immunoaffinity purification. A more recent study reported the identification of more than 29,000 acetylated peptides in the mouse brain of a SARS-CoV-2 infection model (20). To facilitate the analysis of multiple types of PTMs, our group recently reported an integrated method that quantified > 31,000 phosphorylation sites, > 23, 000 thiol oxidation cysteine sites, and > 7,000 lysine acetylation sites from the same sample set in pancreatic beta cells (141). As PTM sample processing is technically challenging and labor-intensive, automation can reduce manual labor and promise better consistency for large-scale studies. Automated sample preparation platforms for phosphorylation, acetylation, and ubiquitination have been developed, using either a magnetic particle handling platform (e.g., KingFisher™ Flex) or a liquid handling platform (e.g., AssayMAP Bravo) (84, 87, 142). The automated pipelines have shown great potential in various fields of study, recently including infectious disease research (123). Making full use of these technical advancements can significantly expand the data obtained from each sample to quantify the temporal dynamics of PTMs over the time course of infection to understand viral replication mechanisms in depth. Importantly, a more comprehensive understanding of how PTM contribute to disease outcomes will likely benefit the development of diagnostic assays and identify novel prophylactic and therapeutic treatment options.

Besides automation, single-cell proteomics allows the analysis of mass limited samples (typical in clinical samples) and can add spatial information to virus-host interaction and viral pathogenesis. However, PTM detection at the single-cell level is still challenging, with the identification of PTMs only on more abundant proteins being reported in current studies (143). Translating single-cell proteomics and PTM-omics into virology studies will likely reveal heterogeneity in the signaling pathways among cells.

Integration of various omics studies including transcriptomics, proteomics, interactomics, metabolomics, and PTM-omics provides a holistic view of virus-host interaction regulated at multiple levels (21, 144, 145). This is especially true for multi-PTM-omics analysis, as it can uncover crosstalk between PTM that control a biological phenotype and might otherwise be hard to identify. With large-scale multi-omics coming of age, standardized approaches for integrative data analysis are still lacking, presenting many challenges (146). For example, varied coverage and depth of each omics data demands tailored analysis. In addition, building the connections between omics data relies on manual curation. With the development of open-source tools (e.g., OmicsNet 2.0 (147)), artificial intelligence is expected to speed up and assist integrative data analysis (148, 149).

Lastly, more PTM-inspired drug development can be expected thanks to the rapidly-growing number of studies performing PTM analysis in models of infectious disease and clinical samples. In addition to pharmaceutical treatments that inhibit pro-viral factors (e.g., CK2 inhibitor Silmitasertib), targeted PTM editing (e.g., via chemically induced proximity (150)) likely offers a more precise therapeutic strategy. A recent study by Oh et. al., demonstrated effective inhibition of SARS-CoV-2 infection by targeted drug delivery to nitrosylate ACE2, preventing viral entry (151). Another study employed preclinical monothiol and dithiol reducing agents P2119 and P2165 to disrupt disulfide pairs in the RBD of SARS-CoV-2 spike glycoprotein, which reduced viral attachment and titer (152). Other strategies can be implied by understanding redox homeostasis during virus replication. As discussed in Section 2.4, oxidative folding of viral proteins plays an important role in the assembly and maturation of progeny viral particles. Disulfide bond formation is catalyzed by PDIs in the endoplasmic reticulum, which maintains an oxidizing environment with a high GSSG/GSH ratio (1:1 to 1:7) (153). As a shift in the ER redox state has a direct impact on PDI activity and the correct folding of viral proteins, one could predict that ER redox perturbation may have antiviral therapeutic potential. In summary, the PTM landscape of both the host and infectious agent provides a spatiotemporally resolved molecular fingerprint of the disease state, making (multi)-PTM-omics one of the most powerful contemporary approaches to understanding the pathophysiology of infectious diseases and developing novel antiviral strategies.
