# Correction: Proteome-wide characterization of PTMs reveals host cell responses to viral infection and identifies putative antiviral drug targets

**DOI:** 10.3389/fimmu.2025.1672766

**Published:** 2025-08-12

**Authors:** Xiaolu Li, Adam Kabza, Ashley N. Ives, Julianne Thiel, Katrina M. Waters, Wei-Jun Qian, Amy C. Sims, Tong Zhang

**Affiliations:** ^1^ Biological Sciences Division, Pacific Northwest National Laboratory, Pacific Northwest National Laboratory, Richland, WA, United States; ^2^ Environmental Molecular Sciences Laboratory, Pacific Northwest National Laboratory, Richland, WA, United States; ^3^ Hanford High School, Richland, WA, United States; ^4^ Nuclear Chemical & Biological Technologies Division, Pacific Northwest National Laboratory, Richland, WA, United States

**Keywords:** PTMs, viral infection, antiviral drug, proteome, phosphorylation, redox, ubiquitination, acetylation

The funder “the Predictive Phenomics Initiative at Pacific Northwest National Laboratory” was erroneously omitted. The correct **Funding** statement appears below:

“The author(s) declare that financial support was received for the research and/or publication of this article. The research described in this paper is part of the Predictive Phenomics Initiative and was conducted under the Laboratory Directed Research and Development Program at Pacific Northwest National Laboratory.”

The original version of this article has been updated.

